# Supplementary material for: Jumbo phage–mediated transduction of genomic islands
Source: Proc Natl Acad Sci U S A. 2025 Oct 28;122(44):e2512465122. doi: 10.1073/pnas.2512465122 (PMC12595487; doi:10.1073/pnas.2512465122)
Supplement: Supplementary file 1 — Appendix 01 (PDF) [file pnas.2512465122.sapp.pdf]

Jumbo phage-mediated transduction of genomic islands

Yansong Zhao[1,*], Yue Ma[2], Christina Vasileiou[1], Andrew D. Farr[1], David W. Rogers[1] & Paul B. Rainey[1,3,*]

[1] Department of Microbial Population Biology, Max Planck Institute for Evolutionary Biology, Plön, Germany

[2] Guest Group Evolutionary Medicine, Max Planck Institute for Evolutionary Biology, Plön, Germany

[3] Laboratory of Biophysics and Evolution, CBI, ESPCI Paris, Université PSL, CNRS, Paris, France

* To whom correspondence may be addressed. Emails: Y.Z. (yansongzhao@evolbio.mpg.de), P.B.R. (rainey@evolbio.mpg.de).

Supplementary Materials and Methods

PCR and gel electrophoresis

All PCR primers used in this study are listed in Table S3. PCR was done with NEB Q5 polymerase, with either extracted DNA or bacterial suspension as templates. Annealing temperatures and extension time was determined as per NEB Tm Calculator (version 1.16.6). Unless otherwise stated, 35 cycles were performed. Gel electrophoresis was performed for 5 µL PCR products with 1x DNA loading dye, in 1% agarose gels.

Plasmid construction

All plasmids used in this study are listed in Table S2. Plasmid pUIsacB (plasmid MPB35522) backbone was amplified by PCR. SBW25 genomic fragments flanking the region of interest, where a mutagenesis, knock-out or knock-in was to be performed, were amplified by PCR. In particular, the Kan^R fragment used to tag I55 was produced by cloning the Kan^R gene from plasmid pMRE-Tn*7*-152 (plasmid MPB27708) followed by a synthetic transcription terminator (CCA ATT ATT GAA CAC CCT TCG GGG TGT TTT TTT GTT TCT GGT CTC CC). Primers were designed with a 5' overhang to facilitate assembly. Backbone and fragments were assembled (with NEBuilder HiFi DNA Assembly Master Mix) and transformed into *E. coli* Top10 competent cells. Successful transformants were selected on LB + Tet plates, and confirmed by PCR and Sanger sequencing across the construct.

Strain construction

Two-step allelic replacement (2SAR)

The method is adapted from Hmelo et al. (1) and extensively described by Farr et al. (2). Briefly, recipient (*P. fluorescens* SBW25), donor (*E. coli* carrying the plasmid used for allelic exchange) and helper (*E. coli* carrying plasmid pRK2013, plasmid MPB35623) strains were cultured overnight until stationary phase. Then, 1 mL recipient culture was heat-shocked at 42 °C for 20 min, and subsequently mixed with 500 µL donor and 500 µL helper culture. The mixture was pelleted and resuspended in 100

µL LB, which was then spotted onto an LB plate for incubation overnight. The cultivated cell layer was then plated out on an LB + nitrofurantoin + Tet plate and incubated overnight to select for trans-conjugates. Single trans-conjugates were then picked and streaked onto TYS10 plates, which were subsequently incubated overnight. Finally, PCR and Sanger sequencing were used to screen for the desired mutants.

Fluorescent tagging

The method is adapted from Schlechter et al. (3). Briefly, recipient (*P. fluorescens* SBW25) and donor (*E. coli* carrying the plasmid pMRE-Tn*7*-152 or pMRE-Tn*7*-155, MPB27708 or MPB27709, respectively) strains were cultured overnight until stationary phase. Then, 1 mL recipient culture was heat-shocked at 42 °C for 20 min, and subsequently mixed with 1 mL donor culture. The mixture was pelleted and resuspended in 100 µL LB, which was then spotted onto an LB plate for incubation overnight. The cultivated cell layer was then plated out on an LB + nitrofurantoin + Kan + 0.1% w/v arabinose plate and incubated overnight to select for trans-conjugates. Single trans-conjugates were picked, and integration of the fluorescent tag was then confirmed by PCR.

Preparation of SBW25-I55 *and* SBW25 *(reconstructed) for assays*

DNA sequencing of the evolved isolate carrying I55 (strain MPB33357) revealed, in addition to presence of the mobile element, two SNPs elsewhere in the genome (see Table S5) that had arisen during the course of serial propagation. Additionally, this isolate also harbored the Tn*7*-152 cassette. While there was no reason to suspect any connection to I55, these SNPs were reverted to the ancestral SBW25 sequence, and Tn*7*-152 removed, by site-directed mutagenesis using the 2SAR strategy described above. Prior to these mutagenesis steps, two independent colonies were picked from the evolved isolate and treated as biological replicates in all subsequent genetic manipulations. The resulting genotype, SBW25-I55 (strain MPB39024/MPB39026) is in principle isogenic with SBW25 (strain MPB29212). However, to be more certain of this fact, the ancestral SBW25 genotype used in the fitness assays and plaque assays was re-generated from SBW25-I55 by removal of the element, giving reconstructed SBW25 (strain MPB39379/MPB39459).

Supplementary Figures

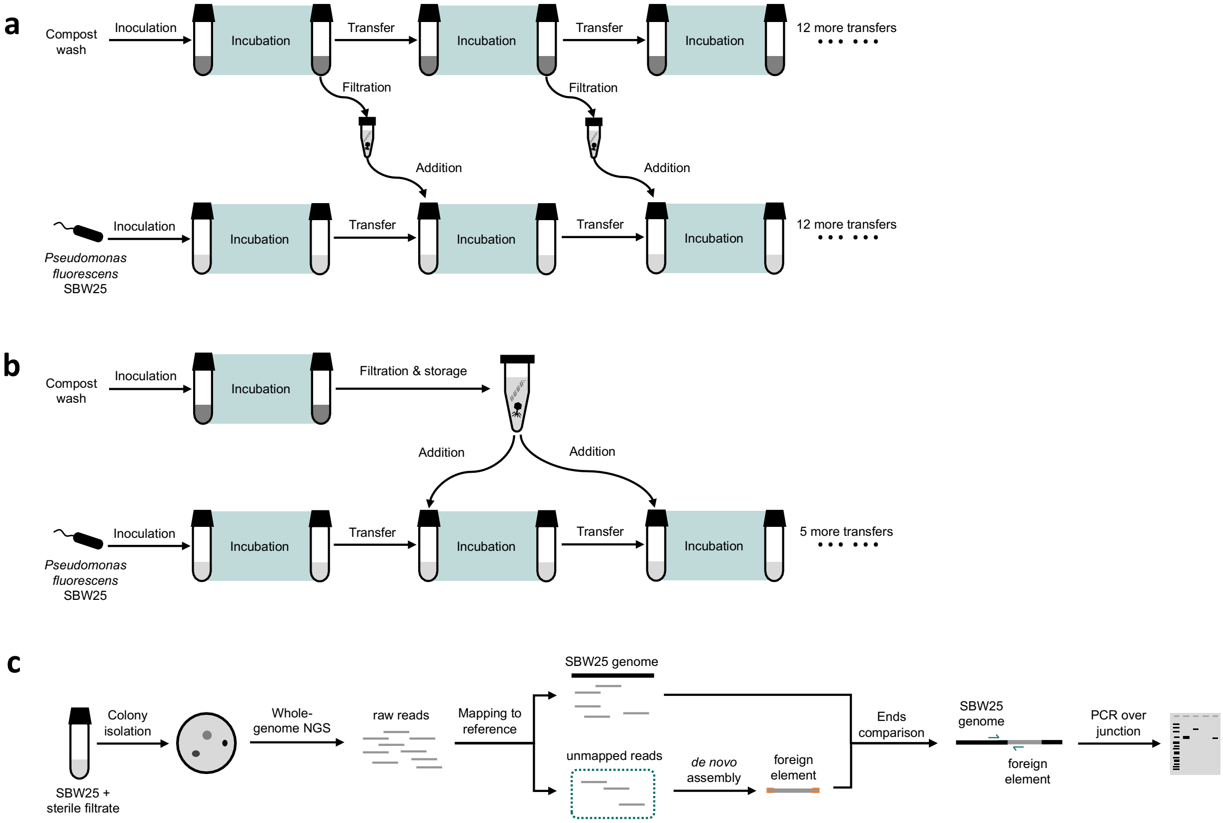

Fig. E1. Experimental evolution regimes and screening pipeline (see Materials and Methods)

a. Schematic of the live-filtrate regime. Here, material was freshly obtained from a compost community (compost C) synchronized with the evolution experiment, with 50 µL filtrate added to each of eight replicate SBW25 populations at every transfer over 14 serial passages.

b. Schematic of the fixed-filtrate regime. Here, filtrate was prepared once from independent compost communities (composts B, C, and D), stored at −80 °C, and added to 10 replicate populations per compost source for seven serial transfers.

c. The pipeline for screening and confirming SBW25 strains carrying foreign elements inserted into the genome.

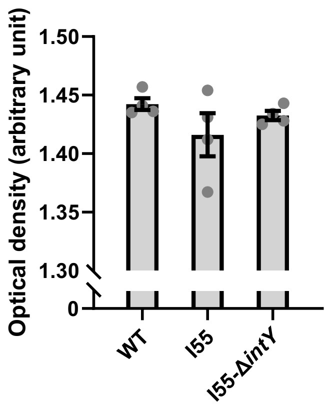

Fig. E2. Measurements of growth of the three strains to be analyzed by PCR

The optical density of overnight LB shaking cultures of SBW25-I55, SBW25-I55Δ*intY* and wild type (WT). Data are mean ± s.d. Brown-Forsythe and Welch ANOVA test was applied to test the difference between the three strains (p = 0.3551).

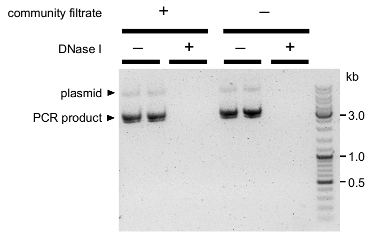

Fig. E3. Testing activity of DNase I in community filtrate

To test the efficacy of the RNase-free DNase I (Norgen) in culture filtrate, the filtrate was spiked with linear PCR products and extracted circular plasmids. The mixture was then treated with the DNase according to the manufacturer's instructions, and visualized by gel electrophoresis. Results showed that the DNase I is active in community filtrate.

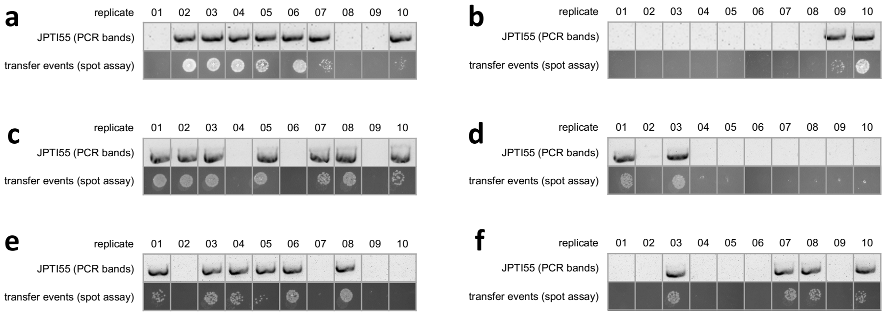

Fig. S1. Full replicates of Fig. 3h.

The example shown in Fig. 3h is panel a here.

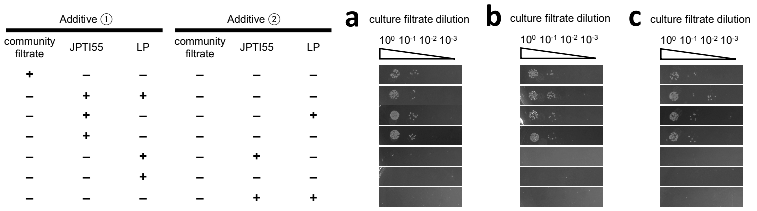

Fig. S2. Full replicates of Fig. 3j.

The example shown in Fig. 3j is panel a here.

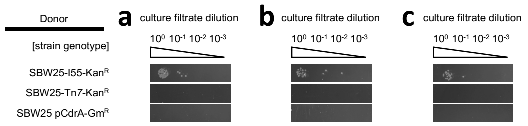

Fig. S3. Full replicates of Fig. 3k.

The example shown in Fig. 3k is panel a here.

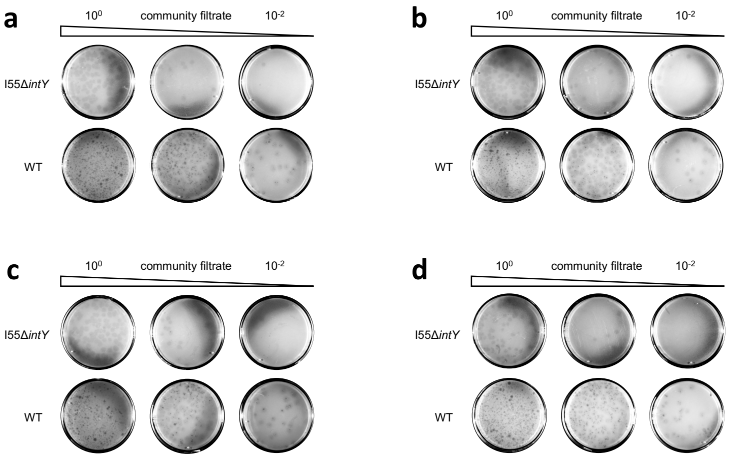

Fig. S4. Full replicates of Fig. 4c.

The example shown in Fig. 4c is panel a here.

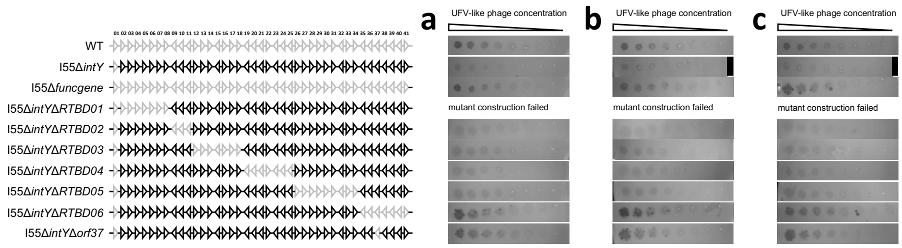

Fig. S5. Full replicates of Fig. 4f.

The example shown in Fig. 4f is panel a here.

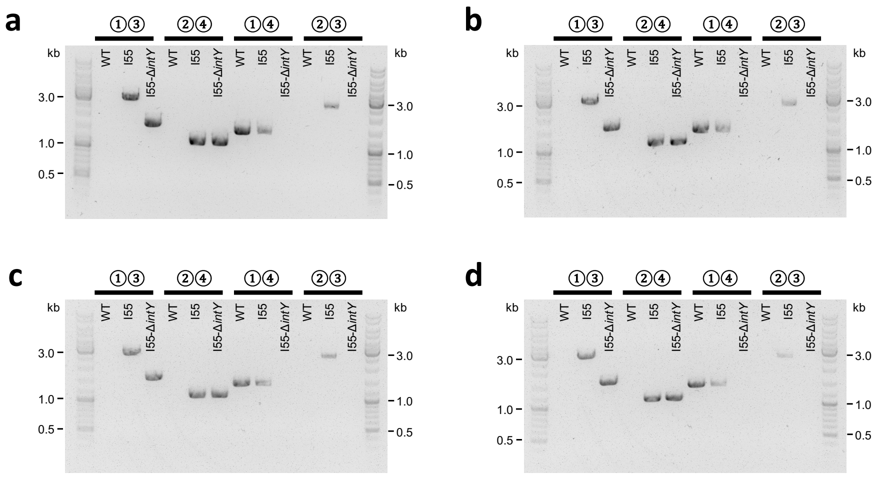

Fig. S6. Full replicates of Fig. 3a.

The example shown in Fig. 3a is panel a here.

Supplementary Tables

Table S1. List of bacterial strains

Number	Name	Genotype	Construction notes
MPB29212	SBW25_Wild_Type	SBW25	Lab stock
MPB29284	Tn7_152_CFC_LB_SBW25	SBW25 *glmS*::Tn*7*-152(GFP, Kan^R)	Conjugation of MPB29212 with MPB27708
MPB29300	Tn7_155_CFC_LB_SBW25	SBW25 *glmS*::Tn*7*-155(RFP, Kan^R)	Conjugation of MPB29212 with MPB27709
MPB38392	SFF_b_GFP_4_iso24_23kb	SBW25 *tmRNA*::I23 *glmS*::Tn*7*-152(GFP, Kan^R) isolate1*	Isolated from fixed-filtrate experiment, compost b, GFP, line 4
MPB38393	SFF_b_GFP_5_iso02_23kb	SBW25 *tmRNA*::I23 *glmS*::Tn*7*-152(GFP, Kan^R) isolate2*	Isolated from fixed-filtrate experiment, compost b, GFP, line 5
MPB38394	SFF_b_GFP_5_iso04_23kb	SBW25 *tmRNA*::I23 *glmS*::Tn*7*-152(GFP, Kan^R) isolate3*	Isolated from fixed-filtrate experiment, compost b, GFP, line 5
MPB38395	SFF_b_GFP_5_iso10_23kb	SBW25 *tmRNA*::I23 *glmS*::Tn*7*-152(GFP, Kan^R) isolate4*	Isolated from fixed-filtrate experiment, compost b, GFP, line 5
MPB38396	SFF_b_GFP_5_iso13_23kb	SBW25 *tmRNA*::I23 *glmS*::Tn*7*-152(GFP, Kan^R) isolate5*	Isolated from fixed-filtrate experiment, compost b, GFP, line 5
MPB38895	SFF_b_RFP_3_iso13_23kb	SBW25 *tmRNA*::I23 *glmS*::Tn*7*-155(RFP, Kan^R) isolate6*	Isolated from fixed-filtrate experiment, compost b, RFP, line 3
MPB38906	SFF_b_RFP_4_iso06_44kb	SBW25 *tmRNA*::I44 *glmS*::Tn*7*-155(RFP, Kan^R) isolate1*	Isolated from fixed-filtrate experiment, compost b, RFP, line 4
MPB38907	SFF_b_RFP_4_iso23_44kb	SBW25 *tmRNA*::I44 *glmS*::Tn*7*-155(RFP, Kan^R) isolate2*	Isolated from fixed-filtrate experiment, compost b, RFP, line 4
MPB33357	SF_G_C_2_T14_iso04	SBW25 *tmRNA*::I55 *glmS*::Tn*7*-152(GFP, Kan^R) isolate1*	Isolated from live-filtrate experiment, compost c, GFP, line 2
MPB38103/MPB38109	Iso04_rev_PFLU4414	SBW25 *tmRNA*::I55 *glmS*::Tn*7*-152(GFP, Kan^R) *pflu3805*SNP	2SAR of two independent colonies of MPB33357 with MPB38033
MPB38572/MPB38574	Iso04_rev_PFLU4414_3805	SBW25 *tmRNA*::I55 *glmS*::Tn*7*-152(GFP, Kan^R)	2SAR of MPB38103/MPB38109 with MPB38391
MPB39024/MPB39026	Iso04_rev_PFLU4414_3805_del_Tn7	SBW25 *tmRNA*::I55	2SAR of MPB38572/MPB38574 with MPB38943
MPB39132/MPB39136	SBW25_I55_Δint	SBW25 *tmRNA*::(I55 Δ*intY*)	2SAR of MPB39024/MPB39026 with MPB38939
MPB39504/MPB39507	SBW25_I55_Δint_Tn7_GFP	SBW25 *tmRNA*::(I55 Δ*intY*) *glmS*::Tn*7*-152(GFP, Kan^R)	Conjugation of MPB39132/MPB39136 with MPB27708
MPB39514/MPB39516	SBW25_I55_Δint_Tn7_RFP	SBW25 *tmRNA*::(I55 Δ*intY*) *glmS*::Tn*7*-152(GFP, Kan^R)	Conjugation of MPB39132/MPB39136 with MPB27709
MPB39379/MPB39459	SBW25_reconstructed	SBW25	2SAR of MPB39132/MPB39136 with MPB39223
MPB39508/MPB39510	SBW25_reconstructed_Tn7_GFP	SBW25 *glmS*::Tn*7*-152(GFP, Kan^R)	Conjugation of MPB39379/MPB39459 with MPB27708
MPB39518/MPB39520	SBW25_reconstructed_Tn7_RFP	SBW25 *glmS*::Tn*7*-155(RFP, Kan^R)	Conjugation of MPB39379/MPB39459 with MPB27709
MPB39465/MPB39467	SBW25_I55_KanR_InsertS1	SBW25 *tmRNA*::(I55 (*orf08-orf09*)::Kan^R)	2SAR of MPB39024/MPB39026 with MPB39269
MPB39565/MPB39568	SBW25_I55_Δint_ KanR_InsertS1	SBW25 *tmRNA*::(I55 Δ*intY* (*orf08-orf09*)::Kan^R)	2SAR of MPB39132/MPB39136 with MPB39269
MPB39500	SBW25-TetR	SBW25 *glmS*::Tn7(AGACTGTTGCGCGCTGGCGGTGGCG, Tet^R)	Lab stock, see https://doi.org/10.1007/s00239-023-10103-6
MPB16791	pCdrA-GFP	SBW25 pCdrA-GFP	Lab stock
MPB40941	55Ele_del_funcgene	SBW25 *tmRNA*::(I55 Δ*funcgene*) **	2SAR of MPB39132 with MPB40717
MPB42170	RTDB_02_del	SBW25 *tmRNA*::(I55 Δ*intY* Δ*RTBD02*) **	2SAR of MPB39132 with MPB41414
MPB41585	RTDB_03_del	SBW25 *tmRNA*::(I55 Δ*intY* Δ*RTBD03*) **	2SAR of MPB39132 with MPB41415
MPB42372	RTDB_04_del	SBW25 *tmRNA*::(I55 Δ*intY* Δ*RTBD04*) **	2SAR of MPB39132 with MPB41416
MPB42031	RTDB_05_del	SBW25 *tmRNA*::(I55 Δ*intY* Δ*RTBD05*) **	2SAR of MPB39132 with MPB41417

MPB41438	RTDB_06_del	SBW25 *tmRNA*::(I55 Δ*intY* Δ*RTBD06*) **	2SAR of MPB39132 with MPB41418
MPB42855	del_I55orf37_MPB42701	SBW25 *tmRNA*::(I55 Δ*intY* Δ*orf37*)	2SAR of MPB39132 with MPB42701
MPB43008	del_I55orf37_MPB42855_GFP	SBW25 *tmRNA*::(I55 Δ*intY* Δ*orf37*) *glmS*::Tn*7*-152(GFP, Kan^{R})	Conjugation of MPB42855 with MPB27708
MPB43010	del_I55orf37_MPB42855_Scarlet (FP)	SBW25 *tmRNA*::(I55 Δ*intY* Δ*orf37*) *glmS*::Tn*7*-155(RFP, Kan^{R})	Conjugation of MPB42855 with MPB27709

* For variations/mutations, see Table S5.

** Regions funcgene, RTBD01, RTBD02, RTBD03, RTBD04, RTBD05, RTBD06 represents the fragments of I55 between and including the following open reading frames: *orf01* to *orf41*, *orf01* to *orf08*, *orf09* to *orf11*, *orf12* to *orf18*, *orf19* to *orf25*, *orf26* to *orf34*, *orf35* to *orf41*.

Table S2. List of *E. coli* plasmids

Number	Name	Description	Source
MPB27708	pMRE-Tn7-152	Plasmid to tag *Pseudomonas* with GFP and Kan^R	Gift from Mitja Remus-Emsermann, see https://www.addgene.org/118566/
MPB27709	pMRE-Tn7-155	Plasmid to tag *Pseudomonas* with RFP and Kan^R	Gift from Mitja Remus-Emsermann, see https://www.addgene.org/118569/
MPB35522	pUIsacB	Empty plasmid for 2-step allelic replacement	Lab stock, see https://www.biorxiv.org/content/10.1101/2024.07.11.603030v1
MPB35623	pRK2013	Helper plasmid for 2-step allelic replacement	Lab stock, see https://www.biorxiv.org/content/10.1101/2024.07.11.603030v1
MPB38033	rev_PFLU4414	Plasmid to revert the SNP in *pflu4414*	this study
MPB38391	rev_PFLU3805	Plasmid to revert the SNP in *pflu3805*	this study
MPB38943	del_Tn7	Plasmid to delete the Tn*7* cassette	this study
MPB38939	del_55kbInt	Plasmid to delete the gene *intY* of I55	this study
MPB39223	del_tmIns	Plasmid to delete any inserts downstream of *tmRNA*	this study
MPB39269	KanR_InsertS1	Plasmid to insert a KanR cassette between *orf08* and *orf09* of I55	this study
MPB41413	RTBD_01_del	Plasmid to delete region RTBD01 of I55	this study
MPB41414	RTBD_02_del	Plasmid to delete region RTBD02 of I55	this study
MPB41415	RTBD_03_del	Plasmid to delete region RTBD03 of I55	this study
MPB41416	RTBD_04_del	Plasmid to delete region RTBD04 of I55	this study
MPB41417	RTBD_05_del	Plasmid to delete region RTBD05 of I55	this study
MPB41418	RTBD_06_del	Plasmid to delete region RTBD06 of I55	this study
MPB42701	del_I55orf37	Plasmid to delete *orf37* of I55	this study

Table S3. List of primers

**Number**	**Name**	**Purpose**	**Sequence**
YZ001	YZ_SBW_5797955F	To detect potential insertions downstream of tmRNA	GTTACCGCTGCGATGTTGAC
YZ002	YZ_SBW_5799313R	To detect potential insertions downstream of tmRNA	GCGTGCTGATGCTGCTAATC
YZ003	YZ_In_1016R	To confirm the presence of I55	GTGGCCAGATCAGGGAAGAC
YZ004	YZ_In_54359F	To confirm the presence of I55	ACACGACACACGAAGAGACC
YZ005	pUIsacB_fwd	To amplify the pUIsacB plasmid backbone	AGCACTACATCAACTGACTA
YZ006	pUIsacB_rev	To amplify the pUIsacB plasmid backbone	CTGAACCAAGATAGCTGTAC
YZ007	2SAR_4880278_Insert_fwd	To construct the plasmid for reverting the SNP in *pflu4414*	GTACAGCTATCTTGGTTCAGGAAATCAACCCATCGCCTG
YZ008	2SAR_4880278_Insert_rev	To construct the plasmid for reverting the SNP in *pflu4414*	TAGTCAGTTGATGTAGTGCTAACGCCTGTCCTATGTGG
YZ009	pUIsacB_seq_fwd	To Sanger sequence the assembled regions of the pUIsacB plasmid	ATTTGTCCTACTCAGGAGAGCGTTC
YZ010	4880278_Insert_4962R	To Sanger sequence the plasmid for reverting the SNP in *pflu4414*	GCTGAGTTCGAATCCCTGCT
YZ011	4880278_Insert_4804F	To Sanger sequence the plasmid for reverting the SNP in *pflu4414*	CTTGCCATGCAGTTGGTCG
YZ012	4880278_Insert_5486R	To Sanger sequence the plasmid for reverting the SNP in *pflu4414*	GGCCGGCGAAATTTTAGAGC
YZ013	4880278_Insert_5275F	To Sanger sequence the plasmid for reverting the SNP in *pflu4414*	CGAATCAACGTGACGCTCAC
YZ014	4880278_Insert_5956R	To Sanger sequence the plasmid for reverting the SNP in *pflu4414*	CGACTGGGGCCGTTTCAT
YZ015	4880278_Insert_5777F	To Sanger sequence the plasmid for reverting the SNP in *pflu4414*	ACACGCTTGATCACCTGACC
YZ016	pUIsacB_seq_rev	To Sanger sequence the assembled regions of the pUIsacB plasmid	AGCGTTCTGAACAAATCCAGATG
YZ084	SBW_23kb_insert_1	To confirm the presence of I23	CCGTTTCTCTTTGGGGTCGA
YZ085	SBW_23kb_insert_2	To confirm the presence of I23	ACTCAAGAGCCTCGCCAAAG
YZ086	2SAR_rev_3805_1	To construct the plasmid for reverting the SNP in *pflu3805*	GTACAGCTATCTTGGTTCAGCCAGCAACGGGTTGTTCTTG
YZ087	2SAR_rev_3805_2	To construct the plasmid for reverting the SNP in *pflu3805*	TAGTCAGTTGATGTAGTGCTCCACTGATCTTGACGCCAGA
YZ088	rev_PFLU3805_4997F	To Sanger sequence the plasmid for reverting the SNP in *pflu3805*	CCTGTTCGCTGAAAATGGCC
YZ089	rev_PFLU3805_5687R	To Sanger sequence the plasmid for reverting the SNP in *pflu3805*	ACACGATGACGACGGTTCTG
YZ091	PFLU4414_Final_Seq_2	To confirm the genotype of mutants after allelic replacement in *pflu4414*	GAACGCATCCACGGCTAATG
YZ092	PFLU3805_Final_Seq_1	To confirm the genotype of mutants after allelic replacement in *pflu3805*	GACACGTCGACCTTCTGGAAG
YZ093	PFLU3805_Final_Seq_2	To confirm the genotype of mutants after allelic replacement in *pflu3805*	CGAGTTGATTGGGGTGGCC
YZ098	PFLU4414_Final_Seq_1_1	To confirm the genotype of mutants after allelic replacement in *pflu4414*	CGCCGACCATGTTCATGATG
YZ102	SBW_44kb_insert_1	To confirm the presence of I44	GCATAGGCTGTGGTCACTGA
YZ103	SBW_44kb_insert_2	To confirm the presence of I44	AGACCAAGGCCGTCAAGAAG
YZ105	2SAR_del_55kbInt_1	To construct the plasmid for deleting the tyrosine integrase (*orf01*) of I55	GTACAGCTATCTTGGTTCAGGTTTAGGATTCGACGCCG
YZ106	2SAR_del_55kbInt_2	To construct the plasmid for deleting the tyrosine integrase (*orf01*) of I55	GAAGTCTCTCGGGTGTATTCCAGTGCTGTTTATG
YZ107	2SAR_del_55kbInt_3	To construct the plasmid for deleting the tyrosine integrase (*orf01*) of I55	TGGAATACACCCGAGAGACTTCAACCAGTGA

YZ108	2SAR_del_55kbInt_4	To construct the plasmid for deleting the tyrosine integrase (*orf01*) of I55	TAGTCAGTTGATGTAGTGCTTCTTCGTTCACCACATTCCG
YZ109	del_55kbInt_4941F	To Sanger sequence the plasmid for deleting the tyrosine integrase (*orf01*) of I55	TGGACGAAGCGGCTAAAACT
YZ110	del_55kbInt_5514R	To Sanger sequence the plasmid for deleting the tyrosine integrase (*orf01*) of I55	TCGCCATTCAGCTGTCCAAT
YZ111	del_55kbInt_Final_Seq_1	To confirm the genotype of mutants after allelic re placement in the tyrosine integrase (*orf01*) of I55	ACGTTAGCTCAGCCGAGGT
YZ112	2SAR_del_Tn7_1	To construct the plasmid for deleting the Tn*7* cassette	GTACAGCTATCTTGGTTCAGTGAGCTATCAGTTGCGCCTG
YZ113	2SAR_del_Tn7_2	To construct the plasmid for deleting the Tn*7* cassette	TAGTCAGTTGATGTAGTGCTAATCCGACCTGACCTTGCTG
YZ114	del_Tn7_4864F	To Sanger sequence the plasmid for deleting the Tn*7* cassette	TTGCGGGTTACGGATGACC
YZ115	del_Tn7_5273F	To Sanger sequence the plasmid for deleting the Tn*7* cassette	GACGTTGGTGGATCAGGGAG
YZ116	del_Tn7_5670F	To Sanger sequence the plasmid for deleting the Tn*7* cassette	CAATGGGCCGTGTTTGAGC
YZ117	del_Tn7_5084R	To Sanger sequence the plasmid for deleting the Tn*7* cassette	TTGGAAGGTGAGCTGCTGTC
YZ118	del_Tn7_5480R	To Sanger sequence the plasmid for deleting the Tn*7* cassette	CTGTCGCCGATCCTCTACAC
YZ119	del_Tn7_5845R	To Sanger sequence the plasmid for deleting the Tn*7* cassette	CAATGGACAGCACCGTGGAA
YZ120	del_Tn7_Final_Seq_1	To confirm the genotype of mutants after allelic replacement in the Tn*7* cassette	ACACCGCCCAACGTGATG
YZ121	del_Tn7_Final_Seq_2	To confirm the genotype of mutants after allelic replacement in the Tn*7* cassette	ACACCCTGTTCGTGACCATC
YZ124	2SAR_KanR_InsertS1_1	To construct the plasmid for inserting an Kan^R cassette between *orf08* and *orf09* of I55	GTACAGCTATCTTGGTTCAGGTCCGCAGAACTGTTGGAG
YZ125	2SAR_KanR_InsertS1_2	To construct the plasmid for inserting an Kan^R cassette between *orf08* and *orf09* of I55	GATTAGCTGTGTCGCTACTG
YZ126	2SAR_KanR_InsertS1_3	To construct the plasmid for inserting an Kan^R cassette between *orf08* and *orf09* of I55	CCGCTCACCGAAGCTGATTA
YZ127	2SAR_KanR_InsertS1_4	To construct the plasmid for inserting an Kan^R cassette between *orf08* and *orf09* of I55	TAGTCAGTTGATGTAGTGCTGGCGACTTCGGTATTCCAG
YZ128	2SAR_KanR_InsertS1_5	To construct the plasmid for inserting an Kan^R cassette between *orf08* and *orf09* of I55	CAGTAGCGACACAGCTAATCGGGAGACCAGAAACAAAAAAACACCC CGAAGGGTGTTCAATAATTGGTCCGCTTCCTTTAGCAGCC
YZ129	2SAR_KanR_InsertS1_6	To construct the plasmid for inserting an Kan^R cassette between *orf08* and *orf09* of I55	TAATCAGCTTCGGTGAGCGGTCCTAGGTGCTCGAGTGG
YZ130	KanR_InsertS1_5008F	To Sanger sequence the plasmid for inserting an Kan^R cassette between *orf08* and *orf09* of I55	TGGCACACGATACCGAAGAC
YZ131	KanR_InsertS1_5508F	To Sanger sequence the plasmid for inserting an Kan^R cassette between *orf08* and *orf09* of I55	AGCGTGCGAGCTCTAAAACA
YZ132	KanR_InsertS1_6011F	To Sanger sequence the plasmid for inserting an Kan^R cassette between *orf08* and *orf09* of I55	ACTCTTCCGAGCAAAGGACG
YZ133	KanR_InsertS1_6662F	To Sanger sequence the plasmid for inserting an Kan^R cassette between *orf08* and *orf09* of I55	GCAGGAAATGGGTCATCGGT
YZ134	KanR_InsertS1_5189R	To Sanger sequence the plasmid for inserting an Kan^R cassette between *orf08* and *orf09* of I55	TTGCAGCAGTGACAAGGACA
YZ135	KanR_InsertS1_5695R	To Sanger sequence the plasmid for inserting an Kan^R cassette between *orf08* and *orf09* of I55	TTGATCTTGGGAGAAGCGGC
YZ136	KanR_InsertS1_6177R	To Sanger sequence the plasmid for inserting an Kan^R cassette between *orf08* and *orf09* of I55	GGACAGCCGGTATAAAGGGA
YZ137	KanR_InsertS1_6853R	To Sanger sequence the plasmid for inserting an Kan^R cassette between *orf08* and *orf09* of I55	TCTCACACGTTCGTTCGACC
YZ144	2SAR_del_tmIns_1	To construct the plasmid for deleting MGEs inserted downstream of *tmRNA*	GTACAGCTATCTTGGTTCAGGAGATCGACACCGCCGTCTC
YZ145	2SAR_del_tmIns_2	To construct the plasmid for deleting MGEs inserted downstream of *tmRNA*	TAGTCAGTTGATGTAGTGCTGCGTGCTGATGCTGCTAATC
YZ146	del_tmIns_5225F	To Sanger sequence the plasmid for deleting MGEs inserted downstream of *tmRNA*	GGGTCGCTGAGGGTTAACTT
YZ147	del_tmIns_5699F	To Sanger sequence the plasmid for deleting MGEs inserted downstream of *tmRNA*	ATGACGCTCGTGTCCAACTT
YZ148	del_tmIns_4886R	To Sanger sequence the plasmid for deleting MGEs inserted downstream of *tmRNA*	CTGTGCAGTGTACGCCAAAC
YZ149	del_tmIns_5575R	To Sanger sequence the plasmid for deleting MGEs inserted downstream of *tmRNA*	GGTACAGACCCTGTTCGTCG

YZ150	del_tmIns_Final_Seq_1	To confirm the genotype of mutants after allelic replacement downstream of *tmRNA*	TTACCGCTGCGATGTTGACT
YZ151	del_tmIns_Final_Seq_2	To confirm the genotype of mutants after allelic replacement downstream of *tmRNA*	GGAGCGTCCGTGAGAGATTC
YZ161	Tn7R-109	To confirm the insertion of Tn7 cassette in SBW25 genome	CAGCATAACTGGACTGATTTCAG
YZ162	SBW25-glmS	To confirm the insertion of Tn7 cassette in SBW25 genome	CACCAAAGCTTTCACCACCCAA
YZ242	Jumbo_Region1_fwd	To screen the presence of the jumbo phage JPTI55	GAAGTCGTACTGGGCACACA
YZ243	Jumbo_Region1_rev	To screen the presence of the jumbo phage JPTI55	GTCACACCACGCACTTCAAC
YZ248	UFV_like_1_fwd	To screen the presence of the UFV-like phage	GACCGTCAGGCATAGGTAGC
YZ249	UFV_like_1_rev	To screen the presence of the UFV-like phage	TTCAGGTCGCGCTCTTTCTT
CV11	RTBD_01_upstream_fwd	To construct the plasmid for deleting RTBD_01 of I55	GTACAGCTATCTTGGTTCAGTAACGTCGCTCCGTCTAAG
CV12	RTBD_01_upstream_rev	To construct the plasmid for deleting RTBD_01 of I55	CTCGAAACCTGGATTGTTATCTTTCTCAGGTTG
CV13	RTBD_01_downstream_fwd	To construct the plasmid for deleting RTBD_01 of I55	ATAACAATCCAGGTTTCGAGAGTAGAAAG
CV14	RTBD_01_downstream_rev	To construct the plasmid for deleting RTBD_01 of I55	TAGTCAGTTGATGTAGTGCTCGTCACACCGAGTTTAAC
CV17	RTBD_02_upstream_fwd	To construct the plasmid for deleting RTBD_02 of I55	GTACAGCTATCTTGGTTCAGTACCGGCAGCTTCGCCTG
CV18	RTBD_02_upstream_rev	To construct the plasmid for deleting RTBD_02 of I55	GTGTCCGAGTATCTTCTCGGCAATCCTTCAGG
CV19	RTBD_02_downstream_fwd	To construct the plasmid for deleting RTBD_02 of I55	CCGAGAAGATACTCGGACACCTCGGCGG
CV20	RTBD_02_downstream_rev	To construct the plasmid for deleting RTBD_02 of I55	TAGTCAGTTGATGTAGTGCTTCGTGAAGTCAGCCGAATTGACG
CV23	RTBD_03_upstream_fwd	To construct the plasmid for deleting RTBD_03 of I55	GTACAGCTATCTTGGTTCAGCTCAAAGCGAAGCCAATAC
CV24	RTBD_03_upstream_rev	To construct the plasmid for deleting RTBD_03 of I55	GGTGAACGGCGGGGATATCTCTCACTGG
CV25	RTBD_03_downstream_fwd	To construct the plasmid for deleting RTBD_03 of I55	AGATATCCCCGCCGTTCACCGAGCACC
CV26	RTBD_03_downstream_rev	To construct the plasmid for deleting RTBD_03 of I55	TAGTCAGTTGATGTAGTGCTCTGACGGTCATAACTCAGAATGTCG
CV29	RTBD_04_upstream_fwd	To construct the plasmid for deleting RTBD_04 of I55	GTACAGCTATCTTGGTTCAGACTCAAGGAGTGGTCCTCG
CV30	RTBD_04_upstream_rev	To construct the plasmid for deleting RTBD_04 of I55	GGAAAGCGTTCCTGCAGCGAGAATCGGTC
CV31	RTBD_04_downstream_fwd	To construct the plasmid for deleting RTBD_04 of I55	TCGCTGCAGGAACGCTTTCCTCAGGTCTG
CV32	RTBD_04_downstream_rev	To construct the plasmid for deleting RTBD_04 of I55	TAGTCAGTTGATGTAGTGCTCAACTTTTCGCCAAGCTG
CV35	RTBD_05_upstream_fwd	To construct the plasmid for deleting RTBD_05 of I55	GTACAGCTATCTTGGTTCAGGGTGTCGTCTCAGCCCCC
CV36	RTBD_05_upstream_rev	To construct the plasmid for deleting RTBD_05 of I55	GTCAGGGTCTGGGGCGAAGCTCCTTGGT
CV37	RTBD_05_downstream_fwd	To construct the plasmid for deleting RTBD_05 of I55	GCTTCGCCCCAGACCCTGACCACCTAGTG
CV38	RTBD_05_downstream_rev	To construct the plasmid for deleting RTBD_05 of I55	TAGTCAGTTGATGTAGTGCT ACAAGATCGGTGTTACGG
CV41	RTBD_06_upstream_fwd	To construct the plasmid for deleting RTBD_06 of I55	GTACAGCTATCTTGGTTCAGGAAGTTCTACCTGGATGCAG
CV42	RTBD_06_upstream_rev	To construct the plasmid for deleting RTBD_06 of I55	CCGAAGCGCTGTCTGATCGGCTTCGTCTC
CV43	RTBD_06_downstream_fwd	To construct the plasmid for deleting RTBD_06 of I55	CCGATCAGACAGCGCTTCGGTTTGACTAAAC
CV44	RTBD_06_downstream_rev	To construct the plasmid for deleting RTBD_06 of I55	TAGTCAGTTGATGTAGTGCTCGAGCGTCATCACGTTTG
CV75	2SAR_del_I55orf37_1	To construct the plasmid for deleting *orf37* of I55	GTACAGCTATCTTGGTTCAGGTCGCGAGCAATGAAGTG
CV76	2SAR_del_I55orf37_2	To construct the plasmid for deleting *orf37* of I55	AATAGCCTCACCTGACTTGCTCCTTTACAGG
CV77	2SAR_del_I55orf37_3	To construct the plasmid for deleting *orf37* of I55	GGAGCAAGTCAGGTGAGGCTATTCCGGTCG

CV78	2SAR_del_I55orf37_4	To construct the plasmid for deleting *orf37* of I55	TAGTCAGTTGATGTAGTGCTCAAGGGGATCGGAGTGAAG

Table S4. Annotations of MGEs I23, I44 and I55

Element	ORF name	Consensus annotation	Minimum coordinate	Maximum coordinate	Length	Direction of transcription	Prokka product	Pfam domains	DefenseFinder prediction
I23	*orf01 (intY)*	tyrosine integrase	186	1442	1257	forward	Prophage integrase IntA	PF13356 (Arm-DNA-bind_3; aa 8-94); PF22022 (Phage_int_M; aa 107-200); PF00589 (Phage_integrase; aa 214-378).	NA
I23	*orf02*	GajA	1631	3436	1806	reverse	ATP-dependent DNA helicase Rep	PF00580 (UvrD-helicase; aa 5-125, 133-239).	Gabija__GajB_1.
I23	*orf03*	GajB	3462	5312	1851	reverse	hypothetical protein	PF13175 (AAA_15; aa 1-72, 310-381); PF20469 (OLD-like_TOPRIM; aa 429-500).	Gabija__GajA.
I23	*orf04*	unannotated	5637	5966	330	forward	hypothetical protein	NA	NA
I23	*orf05*	DUF932	6062	7030	969	forward	hypothetical protein	PF06067 (DUF932; aa 77-302).	NA
I23	*orf06*	YqaJ	7106	8110	1005	forward	hypothetical protein	PF09588 (YqaJ; aa 29-172).	NA
I23	*orf07*	Gp3-like	8201	9151	951	forward	hypothetical protein	PF18897 (Gp3-like; aa 12-206).	NA
I23	*orf08*	RadC	9123	9620	498	forward	hypothetical protein	PF04002 (RadC; aa 44-163).	NA
I23	*orf09*	unannotated	9634	9843	210	forward	hypothetical protein	NA	NA
I23	*orf10*	unannotated	9903	10208	306	forward	hypothetical protein	NA	NA
I23	*orf11*	HTH-containing protein	10195	10542	348	reverse	hypothetical protein	PF01381 (HTH_3; aa 17-71).	NA
I23	*orf12*	unannotated	10681	11034	354	forward	hypothetical protein	NA	NA
I23	*orf13*	unannotated	11155	11973	819	forward	hypothetical protein	NA	NA
I23	*orf14*	DNA topoisomerase	12150	12920	771	reverse	hypothetical protein	PF08378 (NERD; aa 33-145); PF01396 (zf-C4_Topoisom; aa 217-245).	NA
I23	*orf15*	T5orf172 helicase	13022	14206	1185	reverse	hypothetical protein	PF10544 (T5orf172; aa 263-374).	NA
I23	*orf16*	DEAD-like helicase	14199	16265	2067	reverse	hypothetical protein	PF00270 (DEAD; aa 28-193).	NA
I23	*orf17*	unannotated	16268	16372	105	reverse	hypothetical protein	NA	NA
I23	*orf18*	DNA methyltransferase	16369	19131	2763	reverse	hypothetical protein	PF20464 (MmeI_N; aa 1-159); PF20465 (MmeI_hel; aa 170-250); PF20473 (MmeI_Mtase; aa 325-606); PF20466 (MmeI_TRD; aa 629-832); PF20467 (MmeI_C; aa 834-911).	RM_Type_IIG__Type_IIG.
I23	*orf19*	inovirus Gp2	19299	20000	702	reverse	hypothetical protein	PF11726 (Inovirus_Gp2; aa 50-231).	NA
I23	*orf20 (alpA)*	AlpA	20507	20740	234	reverse	hypothetical protein	PF05930 (Phage_AlpA; aa 1-51).	NA
I23	*orf21*	unannotated	20810	22114	1305	reverse	hypothetical protein	NA	NA
I23	*orf22*	unannotated	22346	23020	675	forward	hypothetical protein	NA	NA

I44	*orf01 (intY)*	tyrosine integrase	185	1441	1257	forward	Prophage integrase IntA	PF13356 (Arm-DNA-bind_3; aa 8-94); PF22022 (Phage_int_M; aa 107-200); PF00589 (Phage_integrase; aa 214-377).	NA
I44	*orf02*	type II REase	1609	2364	756	forward	hypothetical protein	PF01844 (HNH; aa 180-235).	RM_Type_II__Type_II_REases
I44	*orf03*	type II Mtase	2467	3531	1065	forward	putative BsuMI modification methylase subunit YdiO	PF00145 (DNA_methylase; aa 4-336).	RM_Type_II__Type_II_MTases
I44	*orf04*	VSR endonuclease	3482	3898	417	reverse	Very short patch repair protein	PF03852 (Vsr; aa 2-64).	NA
I44	*orf05*	DrmC	3916	4554	639	reverse	Cardiolipin synthase B	PF13091 (PLDc_2; aa 67-200).	DISARM__drmC
I44	*orf06*	DrmB	4682	6538	1857	reverse	hypothetical protein	PF09369 (MZB; aa 483-583).	DISARM__drmB
I44	*orf07*	DrmA	6541	10575	4035	reverse	hypothetical protein	NA	DISARM__drmA
I44	*orf08*	unannotated	10579	10908	330	reverse	hypothetical protein	NA	NA
I44	*orf09*	DrmMI	10905	14981	4077	reverse	hypothetical protein	PF07669 (Eco57I; aa 845-931).	DISARM_1__drmMI
I44	*orf10*	DrmD	14978	18103	3126	reverse	RNA polymerase-associated protein RapA	PF00176 (SNF2-rel_dom; aa 134-317); PF00271 (Helicase_C; aa 514-620).	DISARM_1__drmD
I44	*orf11*	MFS transporter	18695	19495	801	reverse	Glycerol-3-phosphate transporter	PF07690 (MFS_1; aa 29-257).	NA
I44	*orf12*	alcohol dehydrogenase	19609	20700	1092	reverse	NAD-dependent methanol dehydrogenase	PF00465 (Fe-ADH; aa 8-351).	NA
I44	*orf13*	avidin	20847	21623	777	reverse	hypothetical protein	PF01382 (Avidin; aa 3-85).	NA
I44	*orf14*	MFS transporter	21691	23055	1365	reverse	Putative niacin/nicotinamide transporter NaiP	PF07690 (MFS_1; aa 46-378).	NA
I44	*orf15*	Asp/Glu/hydantoin racemase	23118	23939	822	reverse	Hydantoin racemase	PF01177 (Asp_Glu_race; aa 20-225).	NA
I44	*orf16*	unannotated	23960	24850	891	reverse	hypothetical protein	NA	NA
I44	*orf17*	metallopeptidase	24907	26292	1386	reverse	Xaa-Pro aminopeptidase	PF05195 (AMP_N; aa 7-123); PF00557 (Peptidase_M24; aa 173-438).	NA
I44	*orf18*	hydantoinase B	26320	27978	1659	reverse	Acetophenone carboxylase delta subunit	PF02538 (Hydantoinase_B; aa 5-522).	NA
I44	*orf19*	hydantoinase A	27992	30076	2085	reverse	Acetophenone carboxylase gamma subunit	PF05378 (Hydant_A_N; aa 9-185); PF01968 (Hydantoinase_A; aa 208-500); PF19278 (Hydant_A_C; aa 514-689).	NA

I44	*orf20*	HTH-type transcriptional regulator	30253	32856	2604	reverse	HTH-type transcriptional regulator MalT	PF00196 (GerE; aa 802-856).	NA
I44	*orf21*	unannotated	33248	33571	324	forward	hypothetical protein	NA	NA
I44	*orf22*	DUF932	33664	34632	969	forward	hypothetical protein	PF06067 (DUF932; aa 77-302).	NA
I44	*orf23*	YqaJ	34707	35711	1005	forward	hypothetical protein	PF09588 (YqaJ; aa 29-172).	NA
I44	*orf24*	Gp3-like	35790	36740	951	forward	hypothetical protein	PF18897 (Gp3-like; aa 12-206).	NA
I44	*orf25*	RadC	36712	37209	498	forward	hypothetical protein	PF04002 (RadC; aa 44-163).	NA
I44	*orf26*	unannotated	37223	37432	210	forward	hypothetical protein	NA	NA
I44	*orf27*	unannotated	37534	37779	246	forward	hypothetical protein	NA	NA
I44	*orf28*	HTH-containing protein	37787	38134	348	reverse	hypothetical protein	PF01381 (HTH_3; aa 17-71).	NA
I44	*orf29*	unannotated	38272	38658	387	forward	hypothetical protein	NA	NA
I44	*orf30*	unannotated	38675	39493	819	forward	hypothetical protein	NA	NA
I44	*orf31*	inovirus Gp2	39957	40916	960	reverse	hypothetical protein	PF11726 (Inovirus_Gp2; aa 149-232).	NA
I44	*orf32 (alpA)*	AlpA	41252	41482	231	reverse	hypothetical protein	PF05930 (Phage_AlpA; aa 1-51).	NA
I44	*orf33*	unannotated	41531	42988	1458	reverse	hypothetical protein	NA	NA
I44	*orf34*	unannotated	43158	43829	672	forward	hypothetical protein	NA	NA
I55	*orf01 (intY)*	tyrosine integrase	185	1441	1257	forward	Prophage integrase IntA	PF13356 (Arm-DNA-bind_3; aa 8-94); PF22022 (Phage_int_M; aa 107-200); PF00589 (Phage_integrase; aa 214-378).	NA
I55	*orf02*	dynamin	2594	4903	2310	forward	hypothetical protein	PF00350 (Dynamin_N; aa 59-226).	Eleos__LeoA
I55	*orf03*	dynamin	4914	7166	2253	forward	hypothetical protein	PF00350 (Dynamin_N; aa 40-177).	NA
I55	*orf04*	unannotated	7235	7738	504	forward	hypothetical protein	NA	NA
I55	*orf05*	unannotated	7735	8307	573	forward	hypothetical protein	NA	NA
I55	*orf06*	unannotated	8349	10991	2643	forward	hypothetical protein	NA	NA
I55	*orf07*	unannotated	11420	12151	732	forward	hypothetical protein	NA	NA
I55	*orf08*	unannotated	12148	13686	1539	forward	hypothetical protein	NA	NA
I55	*orf09*	helicase	14030	16783	2754	reverse	RNA polymerase-associated protein RapA	PF04851 (ResIII; aa 36-245); PF00271 (Helicase_C; aa 755-814).	NA
I55	*orf10*	unannotated	16770	18677	1908	reverse	hypothetical protein	NA	NA
I55	*orf11*	unannotated	18674	20056	1383	reverse	hypothetical protein	NA	NA
I55	*orf12*	unannotated	20681	21103	423	forward	hypothetical protein	NA	NA
I55	*orf13*	DUF1232	21186	21524	339	forward	hypothetical protein	PF06803 (DUF1232; aa 50-84).	NA
I55	*orf14*	unannotated	21652	22302	651	forward	hypothetical protein	NA	NA
I55	*orf15*	unannotated	22316	22447	132	reverse	hypothetical protein	NA	NA

I55	*orf16*	AAA	22605	24695	2091	forward	ATP-dependent zinc metalloprotease FtsH	PF00004 (AAA; aa 255-377, 491-614).	NA
I55	*orf17*	WYL	24791	25813	1023	forward	hypothetical protein	PF13280 (WYL; aa 156-330).	WYL_I_II_III_IV_V_VI_4
I55	*orf18*	unannotated	25859	26395	537	forward	hypothetical protein	NA	NA
I55	*orf19*	Sir2	26521	27174	654	reverse	NAD-dependent protein deacylase	PF02146 (SIR2; aa 20-196).	NA
I55	*orf20*	unannotated	27411	27668	258	reverse	hypothetical protein	NA	NA
I55	*orf21*	Sel1	27658	28299	642	reverse	hypothetical protein	PF08238 (Sel1; aa 96-127, 133-166, 171-200).	NA
I55	*orf22*	unannotated	28421	28585	165	forward	hypothetical protein	NA	NA
I55	*orf23*	RecD	28573	30666	2094	reverse	RecBCD enzyme subunit RecD	PF21185 (RecD_N; aa 40-153); PF13245 (AAA_19; aa 205-357); PF13538 (UvrD_C_2; aa 615-662).	NA
I55	*orf24*	RecB	30663	34337	3675	reverse	RecBCD enzyme subunit RecB	PF00580 (UvrD-helicase; aa 13-452); PF13361 (UvrD_C; aa 459-660); PF13361 (UvrD_C; aa 710-848); PF12705 (PDDEXK_1; aa 1072-1214).	Gabija__GajB_2
I55	*orf25*	RecC	34334	37804	3471	reverse	RecBCD enzyme subunit RecC	PF04257 (Exonuc_V_gamma; aa 11-360); PF17946 (RecC_C; aa 835-1081).	NA
I55	*orf26*	unannotated	38042	38371	330	forward	hypothetical protein	NA	NA
I55	*orf27*	DUF932	38462	39430	969	forward	hypothetical protein	PF06067 (DUF932; aa 77-302).	NA
I55	*orf28*	YqaJ	39513	40514	1002	forward	hypothetical protein	PF09588 (YqaJ; aa 28-171).	NA
I55	*orf29*	Gp3-like	40605	41498	894	forward	hypothetical protein	PF18897 (Gp3-like; aa 12-206).	NA
I55	*orf30*	unannotated	41554	41742	189	forward	hypothetical protein	NA	NA
I55	*orf31*	unannotated	41803	42126	324	forward	hypothetical protein	NA	NA
I55	*orf32*	HTH-containing protein	42136	42483	348	reverse	hypothetical protein	PF01381 (HTH_3; aa 17-71).	NA
I55	*orf33*	unannotated	42622	42975	354	forward	hypothetical protein	NA	NA
I55	*orf34*	unannotated	43189	43812	624	forward	hypothetical protein	NA	NA
I55	*orf35*	MUG113	43980	45188	1209	reverse	hypothetical protein	PF13455 (MUG113; aa 285-373).	NA
I55	*orf36*	DEAD-like helicase	45181	47247	2067	reverse	hypothetical protein	PF00270 (DEAD; aa 28-193).	NA
I55	*orf37*	DNA methyltransferase	47349	50105	2757	reverse	hypothetical protein	PF20464 (MmeI_N; aa 1-159); PF20465 (MmeI_hel; aa 170-250); PF20473 (MmeI_Mtase; aa 325-605); PF20466 (MmeI_TRD; aa 627-826); PF20467 (MmeI_C; aa 827-909).	RM_Type_IIG__Type_IIG
I55	*orf38*	inovirus Gp2	50350	51321	972	reverse	hypothetical protein	NA	NA
I55	*orf39 (alpA)*	AlpA	51661	51888	228	reverse	hypothetical protein	PF05930 (Phage_AlpA; aa 1-51).	NA
I55	*orf40*	unannotated	51950	53260	1311	reverse	hypothetical protein	NA	NA

I55	*orf41*	unannotated	53417	54289	873	forward	hypothetical protein	NA	NA

- **ORF name**: This is the name given to a gene on an MGE. On each MGE, genes are named serially and consecutively from 5'-end (near tyrosine integrase) to 3'-end in the format *orf*#, where # is a two-digit number. The tyrosine integrase always has #=01, the genes following it have #=02, 03, 04, etc. For some well-recognisable genes, common gene names are indicated inside round brackets following *orf*#.
- **Consensus annotation**: This is the annotation given to a gene after manual checks on Prokka and Pfam predictions. Genes are called "unannotated" when neither predictors find results.
- **Minimum coordinate**: This is the position of the first nucleotide of a gene. For each MGE, the nucleotides are numbered from the 5'-end, with the first nucleotide immediately downstream of the 14 bp repeat (near the 3'-end of *tmRNA*) labelled as 1.
- **Maximum coordinate**: This is the position of the last nucleotide of a gene.
- **Length**: This is the number of nucleotides in a gene, and is equal to Maximum coordinate - Minimum coordinate + 1.
- **Direction of transcription**: This is the direction of transcription of a gene. Genes labelled as "forward" are transcribed from the 5'-end to the 3'-end along the annotated strand (i.e., the strand where the tyrosine integrase is at the 5'-end). Genes labelled as "forward" are transcribed from the 5'-end to the 3'-end along the other stand.
- **Prokka product**: This is the protein product predicted by Prokka in KBase. Proteins without predictions are labelled as "hypothetical protein".
- **Pfam domains**: This is the protein domain(s) predicted by Pfam in InterProScan. For each predicted domain, the Pfam Id is listed, with the corresponding Pfam name and mapped region (given by the interval of amino acids along the amino acid sequence) given in round brackets. For proteins with multiple predicted domains, all domains are listed and separated by semicolons. Proteins with no predicted domains are labelled as "NA".
- **DefenseFinder prediction**: This is the gene name of putative phage defence genes predicted by DenfenseFinder. Genes not predicted to be involved in phage defence activities are labelled as "NA".

Table S5. Mutations detected in isolates with MGEs.

Strain number	Genotype	Gene locus	Genome coordinate	Type of mutation	Change	CDS position	Effect on protein
MPB38392	SBW25 *tmRNA*::I23 *glmS*::Tn*7*-152(GFP, Kan^{R}) isolate1	*pflu4414*	4878944-4878945	insertion (tandem repeat)	T(2) -> T(3)	1489-1490	frameshift
MPB38393	SBW25 *tmRNA*::I23 *glmS*::Tn*7*-152(GFP, Kan^{R}) isolate2	*pflu4414 - pflu4415*	4880381-4880570	deletion	del 190 bp	*pflu4414*: 1-53; *pflu4415*: 664-789	*pflu4415*: truncation; *pflu4414*: no translation
MPB38394	SBW25 *tmRNA*::I23 *glmS*::Tn*7*-152(GFP, Kan^{R}) isolate3	*pflu4414*	4880259	SNP (transition)	G -> A	175	truncation
MPB38395	SBW25 *tmRNA*::I23 *glmS*::Tn*7*-152(GFP, Kan^{R}) isolate4	*pflu3022*	3295100-3295889	deletion	del 790 bp	387-1176	truncation
		pflu4414	4879083-4879084	insertion (tandem repeat)	T(5) -> T(6)	1350-1351	frameshift
MPB38396	SBW25 *tmRNA*::I23 *glmS*::Tn*7*-152(GFP, Kan^{R}) isolate5	*pflu4414 - pflu4415*	4880381-4880570	deletion	del 190 bp	*pflu4414*: 1-53; *pflu4415*: 664-789	*pflu4415*: truncation; *pflu4414*: no translation
MPB38895	SBW25 *tmRNA*::I23 *glmS*::Tn*7*-155(RFP, Kan^{R}) isolate6	None	-	-	-	-	-
MPB38906	SBW25 *tmRNA*::I44 *glmS*::Tn*7*-155(RFP, Kan^{R}) isolate1	*pflu4744*	5217912	SNP (transition)	G -> A	116	substitution (S39N)
MPB38907	SBW25 *tmRNA*::I44 *glmS*::Tn*7*-155(RFP, Kan^{R}) isolate2	*pflu4744*	5217912	SNP (transition)	G -> A	116	substitution (S39N)
MPB33357	SBW25 *tmRNA*::I55 *glmS*::Tn*7*-152(GFP, Kan^{R}) isolate1	*pflu3805*	4199776	SNP (transversion)	A -> T	33	substitution (N11K)
		pflu4414	4880277-4880278	insertion (tandem repeat)	C(5) -> C(6)	157-158	frameshift

Table S6. Rank abundance of genes carried by similar MGEs

Domains	COG category	Description	Counts
Phage_integrase	L	Belongs to the 'phage' integrase family	111
AlpA	K	transcriptional regulator	109
Inovirus_Gp2	S	Inovirus Gp2	101
WYL	K	regulation of single-species biofilm formation	87
DUF932	S	Domain of unknown function (DUF932)	80
YqaJ	L	COG5377 Phage-related protein	78
RadC	L	RadC-like JAB domain	66
DUF1524,DUF262	S	Protein of unknown function DUF262	42
HsdM_N,N6_Mtase	V	Type I restriction-modification system methyltransferase subunit	40
Methylase_S	V	PFAM Type I restriction modification DNA specificity domain	31
N6_Mtase	V	Type II restriction enzyme, methylase	24
DUF1788	S	Domain of unknown function (DUF1788)	23
AAA_5	V	GTPase subunit of restriction endonuclease	20
Dynamin_N	T	ATPase. Has a role at an early stage in the morphogenesis of the spore coat	20
HTH_3,HTH_31	K	sequence-specific DNA binding	20
MMR_HSR1	P	Dynamin family	17
ADP_ribosyl_GH	O	ADP-ribosylglycohydrolase	16
DNA_methylase	H	Belongs to the class I-like SAM-binding methyltransferase superfamily. C5-methyltransferase family	16
DUF262	S	Protein of unknown function DUF262	16
Helicase_C,SNF2_N	KL	Transcription regulator that activates transcription by stimulating RNA polymerase (RNAP) recycling in case of stress conditions such as supercoiled DNA or high salt concentrations. Probably acts by releasing the RNAP, when it is trapped or immobilized on tightly supercoiled DNA. Does not activate transcription on linear DNA. Probably not involved in DNA repair	15
Vsr	L	May nick specific sequences that contain T G mispairs resulting from m5C-deamination	15
EcoR124_C,HSDR_N,ResIII	L	Subunit R is required for both nuclease and ATPase activities, but not for modification	14
Lon_2,Lon_C	O	Putative ATP-dependent Lon protease	14
PDDEXK_1,UvrD-helicase,UvrD_C	L	A helicase nuclease that prepares dsDNA breaks (DSB) for recombinational DNA repair. Binds to DSBs and unwinds DNA via a highly rapid and processive ATP-dependent bidirectional helicase activity. Unwinds dsDNA until it encounters a Chi (crossover hotspot instigator) sequence from the 3' direction. Cuts ssDNA a few nucleotides 3' to the Chi site. The properties and activities of the enzyme are changed at Chi. The Chi-altered holoenzyme produces a long 3'-ssDNA overhang and facilitates RecA-binding to the ssDNA for homologous DNA recombination and repair. Holoenzyme degrades any linearized DNA that is unable to undergo homologous recombination. In the holoenzyme this subunit contributes ATPase, 3'-5' helicase, exonuclease activity and loads RecA onto ssDNA	14
Helicase_C	KL	Helicase conserved C-terminal domain	13
HTH_1,LysR_substrate	K	transcriptional regulator	13

McrBC	V	McrBC 5-methylcytosine restriction system component	13
HSDR_N,ResIII	L	Subunit R is required for both nuclease and ATPase activities, but not for modification	12
HTH_17	K	TIGRFAM DNA binding domain, excisionase family	12
DUF1998	S	Domain of unknown function (DUF1998)	11
PglZ	H	PglZ domain	11
Ank_2,Ank_4,Ank_5	G	response to abiotic stimulus	10
N6_N4_Mtase	H	DNA methylase N-4 N-6	10
ResIII	KL	Type III restriction enzyme, res subunit	10
DEAD,DUF1998,Helicase_C	L	COG1205 Distinct helicase family with a unique C-terminal domain including a metal-binding cysteine cluster	9
DUF1819	S	Putative inner membrane protein (DUF1819)	9
DUF45	S	Metal-dependent hydrolase	9
HTH_Tnp_1	L	Transposase and inactivated derivatives	9
Lon_2,Lon_C,MIT_C	O	ATP-dependent Lon-type protease	9
PLDc_2	I	phospholipase d transphosphatidylase	9
AAA_15	L	AAA ATPase domain	8
BPD_transp_1	P	Binding-protein-dependent transport system inner membrane component	8
DNA_processg_A	LU	DNA recombination-mediator protein A	8
DUF697,MMR_HSR1	D	50S ribosome-binding GTPase	8
Transp_cyt_pur	FH	Permease for cytosine/purines, uracil, thiamine, allantoin	8
AAA_15,AAA_21,AAA_23	T	AAA domain, putative AbiEii toxin, Type IV TA system	7
DEAD,Helicase_C,RecQ_Zn_bind	L	DNA helicase	7
Eco57I,N6_Mtase	V	COG1002 Type II restriction enzyme, methylase subunits	7
AAA_11,AAA_12,AAA_30,PDDEXK_1,RNase_H_2	L	RNase_H superfamily	6
AAA_19,AAA_30,UvrD_C_2	L	A helicase nuclease that prepares dsDNA breaks (DSB) for recombinational DNA repair. Binds to DSBs and unwinds DNA via a highly rapid and processive ATP-dependent bidirectional helicase activity. Unwinds dsDNA until it encounters a Chi (crossover hotspot instigator) sequence from the 3' direction. Cuts ssDNA a few nucleotides 3' to the Chi site. The properties and activities of the enzyme are changed at Chi. The Chi-altered holoenzyme produces a long 3'-ssDNA overhang and facilitates RecA-binding to the ssDNA for homologous DNA recombination and repair. Holoenzyme degrades any linearized DNA that is unable to undergo homologous recombination. In the holoenzyme this subunit has ssDNA-dependent ATPase and 5'-3' helicase activity. When added to pre-assembled RecBC greatly stimulates nuclease activity and augments holoenzyme processivity. Negatively regulates the RecA-loading ability of RecBCD	6
AAA_23	L	ATPase involved in DNA repair	6
ABC_tran,DUF726,Dynamin_N	S	ATPase. Has a role at an early stage in the morphogenesis of the spore coat	6
CxxCxxCC	S	PFAM Uncharacterised protein family (UPF0153)	6
DUF2357,PDDEXK_7	S	PD-(D/E)XK nuclease superfamily	6

DUF445	S	membrane	6
EH_Signature	S	EH_Signature domain	6
Exonuc_V_gamma	L	A helicase nuclease that prepares dsDNA breaks (DSB) for recombinational DNA repair. Binds to DSBs and unwinds DNA via a highly rapid and processive ATP-dependent bidirectional helicase activity. Unwinds dsDNA until it encounters a Chi (crossover hotspot instigator) sequence from the 3' direction. Cuts ssDNA a few nucleotides 3' to the Chi site. The properties and activities of the enzyme are changed at Chi. The Chi-altered holoenzyme produces a long 3'-ssDNA overhang and facilitates RecA-binding to the ssDNA for homologous DNA recombination and repair. Holoenzyme degrades any linearized DNA that is unable to undergo homologous recombination. In the holoenzyme this subunit recognizes the wild- type Chi sequence, and when added to isolated RecB increases its ATP-dependent helicase processivity	6
Helicase_C,Mrr_cat,SNF2_N, SWIM	KL	COG0553 Superfamily II DNA RNA helicases, SNF2 family	6
PDDEXK_1	L	PD-(D/E)XK nuclease superfamily	6
Amidohydro_3	F	cytosine deaminase	5
DDE_Tnp_1,DDE_Tnp_1_6,DUF772	L	Transposase DDE domain	5
DUF1232	S	Protein of unknown function (DUF1232)	5
DUF3987	T	Protein of unknown function (DUF3987)	5
Helicase_C,ResIII,SNF2_N	L	PFAM Helicase conserved C-terminal domain	5
HNH_2	V	restriction endonuclease	5
Hydant_A_N,Hydantoinase_A	EQ	N-methylhydantoinase A acetone carboxylase, beta subunit	5
Hydantoinase_B	EQ	Hydantoinase B/oxoprolinase	5
Isochorismatase	Q	Isochorismatase family	5
MFS_1	EGP	Major Facilitator Superfamily	5
MFS_1,Sugar_tr	EGP	Major facilitator superfamily	5
MotB_plug,OmpA	N	Membrane MotB of proton-channel complex MotA/MotB	5
PDDEXK_7	S	PD-(D/E)XK nuclease superfamily	5
UvrD-helicase,UvrD_C,UvrD_C_2	L	Uvrd rep helicase	5
Virulence_RhuM	S	COG3943 Virulence protein	5
AAA	O	COG0464 ATPases of the AAA class	4
AAA_13	L	AAA domain	4
AAA_5,DUF3578	V	Domain of unknown function (DUF3578)	4
ABC_tran,TOBE_2	P	Belongs to the ABC transporter superfamily	4
DAO	E	FAD dependent oxidoreductase	4
DUF2950	N	Protein of unknown function (DUF2950)	4
DUF3077	S	Protein of unknown function (DUF3077)	4
DUF4357	S	Domain of unknown function (DUF4357)	4

Fer2_4	C	2Fe-2S iron-sulfur cluster binding domain	4
Fer2_BFD,Pyr_redox_2	C	FAD-dependent pyridine nucleotide-disulphide oxidoreductase	4
Helicase_C,ResIII	L	Type III restriction protein res subunit	4
His_Phos_1,NUDIX	L	nUDIX hydrolase	4
HTH_30,PucR	QT	transcriptional regulator	4
Ketoacyl-synt_C,ketoacyl-synt	IQ	catalyzes a condensation reaction in fatty acid biosynthesis addition of an acyl acceptor of two carbons from malonyl-ACP	4
Pkinase	KLT	Protein tyrosine kinase	4
Ribonuc_L-PSP	J	Endoribonuclease L-PSP	4
SBP_bac_6,SBP_bac_8	E	Bacterial extracellular solute-binding protein	4
AAA_11,AAA_12,NERD,Pkinase	L	AAA domain	3
Arm-DNA-bind_3,Phage_integrase	L	Belongs to the 'phage' integrase family	3
DDE_Tnp_IS66,DDE_Tnp_IS66_C,LZ_Tnp_IS66,zf-IS66	S	zinc-finger binding domain of transposase IS66	3
DEAD,Helicase_C	L	helicase superfamily c-terminal domain	3
DJ-1_PfpI,HTH_18	K	COG4977 Transcriptional regulator containing an amidase domain and an AraC-type DNA-binding HTH domain	3
DUF3387,HSDR_N,ResIII	F	Subunit R is required for both nuclease and ATPase activities, but not for modification	3
Helicase_C,PLDc_2,SNF2_N	L	SNF2 family N-terminal domain	3
HTH_21,rve	L	Transposase and inactivated derivatives	3
Mrr_cat	V	Restriction endonuclease	3
NA37	S	37-kD nucleoid-associated bacterial protein	3
RelA_SpoT	S	Region found in RelA / SpoT proteins	3
RHH_1	S	Ribbon-helix-helix protein, copG family	3
Sel1	S	Sel1-like repeats.	3
TnpB_IS66	L	Transposase	3
AAA_15,AAA_21,ABC_tran	L	(ABC) transporter	2
AAA_19,NERD,UvrD_C_2	L	COG0210 Superfamily I DNA and RNA helicases	2
AAA_19,UvrD-helicase	L	UvrD/REP helicase N-terminal domain	2
AAA_19,UvrD-helicase,UvrD_C,UvrD_C_2	L	UvrD/REP helicase N-terminal domain	2
Acyl-CoA_dh_1,Acyl-CoA_dh_M,Acyl-CoA_dh_N	I	Acyl-CoA dehydrogenase, middle domain	2
adh_short_C2	IQ	PFAM Short-chain dehydrogenase reductase SDR	2
AIPR	S	AIPR protein	2
Aldedh	C	Aldehyde dehydrogenase family	2

CoA_transf_3	C	CoA-transferase family III	2
DUF1837	S	Domain of unknown function (DUF1837)	2
DUF3223	S	Protein of unknown function (DUF3223)	2
DUF3883	O	Molecular chaperone. Has ATPase activity	2
DUF4868	S	Domain of unknown function (DUF4868)	2
DUF726	S	Protein of unknown function (DUF726)	2
EcoEI_R_C,HSDR_N,Helicase_C,ResIII	L	EcoEI R protein C-terminal	2
GAF,HATPase_c,HisKA,PAS_2,PHY	T	PAS fold	2
HATPase_c_3	L	Histidine kinase-, DNA gyrase B-, and HSP90-like ATPase	2
HNH	L	HNH nucleases	2
HpcH_HpaI	G	HpcH/HpaI aldolase/citrate lyase family	2
HTH_29,rve,rve_2,rve_3	L	COG2801 Transposase and inactivated derivatives	2
HTH_31	K	Helix-turn-helix XRE-family like proteins	2
HxlR	K	transcriptional regulator	2
MaoC_dehydrat_N,MaoC_dehydratas	I	N-terminal half of MaoC dehydratase	2
MUG113,T5orf172	P	T5orf172	2
NmrA	GM	NmrA family	2
PDDEXK_1,Peptidase_M78,UvrD-helicase,UvrD_C	L	Unwinds DNA duplexes with 3' to 5' polarity with respect to the bound strand and initiates unwinding most effectively when a single-stranded region is present	2
Plug,TonB_dep_Rec	P	TonB dependent receptor	2
PRiA4_ORF3	L	Plasmid pRiA4b ORF-3-like protein	2
RES	S	RES	2
Response_reg	T	response regulator	2
SIR2_2	F	SIR2-like domain	2
UvrD_C_2	L	UvrD-like helicase C-terminal domain	2
3HCDH,3HCDH_N	I	3-hydroxyacyl-coa dehydrogenase	1
AAA_11,AAA_12,DUF3320,DUF4011,DUF559	L	AAA domain	1
AAA_16,GAF,GAF_2,HATPase_c,HisKA,PAS_3,Pkinase	T	Histidine kinase	1
AAA_18,AAA_33,CPT	S	AAA domain	1
AAA_19,UvrD-helicase,UvrD_C	L	DNA helicase	1
AAA_21	S	AAA domain, putative AbiEii toxin, Type IV TA system	1

AAA_30,UvrD_C_2	L	PIF1-like helicase	1
Abhydrolase_1,Abhydrolase_6	I	Alpha beta hydrolase	1
Abhydrolase_1,Hydrolase_4	I	hydrolases or acyltransferases (alpha beta hydrolase superfamily)	1
ABM	S	Antibiotic biosynthesis monooxygenase	1
AcetylCoA_hyd_C,AcetylCoA_hydro	C	acetyl-CoA hydrolase	1
Acetyltransf_1,Acetyltransf_10,tRNA_m1G_MT	K	acetyltransferase	1
ACR_tran	V	TIGRFAM transporter, hydrophobe amphiphile efflux-1 (HAE1) family	1
Acyl-CoA_dh_1,Acyl-CoA_dh_N	I	acyl-CoA dehydrogenase	1
Adenylsucc_synt	F	Adenylosuccinate synthetase	1
ADH_N,ADH_zinc_N	C	Zinc-binding dehydrogenase	1
ADH_N,ADH_zinc_N,ADH_zinc_N_2	C	alcohol dehydrogenase	1
ADH_N_2,ADH_zinc_N	S	N-terminal domain of oxidoreductase	1
adh_short	S	Belongs to the short-chain dehydrogenases reductases (SDR) family	1
adh_short,adh_short_C2	IQ	KR domain	1
Amidohydro_2	S	Amidohydrolase	1
AMP_N,Peptidase_M24	E	Aminopeptidase P, N-terminal domain	1
ApbA,ApbA_C	H	Ketopantoate reductase PanE/ApbA C terminal	1
AraC_binding,HTH_18	K	Transcriptional regulator	1
Arginase	E	Belongs to the arginase family	1
Biotin_lipoyl_2,HlyD_3,HlyD_D23	M	Belongs to the membrane fusion protein (MFP) (TC 8.A.1) family	1
CitMHS	C	TIGRFAM citrate H symporter, CitMHS family	1
Cytochrome_CBB3	C	photosynthesis	1
DDE_3,HTH_23,HTH_32,HTH_33	L	COG3335 Transposase and inactivated derivatives	1
DDE_Tnp_1	L	PFAM Protein	1
DDE_Tnp_1,DUF772	L	COG3039 Transposase and inactivated derivatives, IS5 family	1
DDE_Tnp_1,Methylase_S	V	type I restriction modification DNA specificity domain	1
DEAD,Helicase_C,Pribosyltran,RecQ_Zn_bind,UvrD-helicase,UvrD_C	L	helicase superfamily c-terminal domain	1
DJ-1_PfpI	S	DJ-1/PfpI family	1

DoxX_2	S	DoxX-like family	1
DUF1302	M	Protein of unknown function (DUF1302)	1
DUF2236	S	Uncharacterized protein conserved in bacteria (DUF2236)	1
DUF2867	S	Protein of unknown function (DUF2867)	1
DUF2938	S	Protein of unknown function (DUF2938)	1
DUF2946	S	Protein of unknown function (DUF2946)	1
DUF4113,IMS,IMS_C,IMS_HHH	L	DNA polymerase	1
DUF4420	S	Putative PD-(D/E)XK family member, (DUF4420)	1
DUF928,SIR2_2	F	5-carbamoylmethyl uridine residue modification	1
Dynamin_N,MMR_HSR1,Patatin	M	Esterase of the alpha-beta hydrolase superfamily	1
EcoEI_R_C,Helicase_C,ResIII	L	EcoEI R protein C-terminal	1
EcoRII-C	L	EcoRII C terminal	1
ETF	C	Electron transfer flavoprotein	1
ETF,ETF_alpha	C	Electron transfer flavoprotein	1
ETF_QO,FAD_binding_2,NAD_binding_8,Thi4	C	Electron transfer flavoprotein-ubiquinone oxidoreductase	1
Fe-ADH	C	Iron-containing alcohol dehydrogenase	1
Fer4_12,Fer4_14,Radical_SAM	S	Radical SAM superfamily	1
Fer4_12,Radical_SAM	S	Radical SAM superfamily	1
Flg_new,LRR_5,LRR_6	S	regulation of response to stimulus	1
GAF,GAF_2,GGDEF,PAS_3	T	GAF PAS GGDEF domain-containing protein	1
GerE	K	helix_turn_helix, Lux Regulon	1
GerE,Response_reg,Trans_reg_C	K	response regulator	1
GntR,UTRA	K	UTRA	1
Histone_HNS	-	-	1
HTH_11,WYL	K	HTH domain	1
HTH_18	K	AraC Family	1
HTH_20	K	transcriptional regulator	1
HTH_21,rve,rve_3	L	PFAM Integrase catalytic region	1
HTH_23,HTH_24,rve	L	Transposase and inactivated derivatives	1
HTH_26	K	transcriptional regulator	1
HTH_3,Peptidase_M78	K	IrrE N-terminal-like domain	1

HTH_IclR,IclR	K	transcriptional regulator	1
Integrase_1,Phage_int_SAM_2	L	Belongs to the 'phage' integrase family	1
Lactamase_B	S	Metallo-beta-lactamase superfamily	1
Lumazine_bd_2	S	Putative lumazine-binding	1
LysR_substrate	K	LysR substrate binding domain	1
M20_dimer,Peptidase_M20	S	Peptidase dimerisation domain	1
MaoC_dehydratas	I	MaoC like domain	1
MS_channel,cNMP_binding	MT	mechanosensitive ion channel	1
N6_N4_Mtase,TypeIII_RM_meth	L	DNA methylase	1
NAD_binding_10,NmrA	GM	NmrA-like family	1
NERD,zf-C4_Topoisom	L	COG0551 Zn-finger domain associated with topoisomerase type I	1
NUDIX	L	NUDIX domain	1
OEP	M	RND efflux system, outer membrane lipoprotein	1
OmpA	N	Flagellar Motor Protein	1
OsmC	O	response to oxidative stress	1
Oxidored_FMN	C	PFAM NADH flavin oxidoreductase NADH oxidase	1
PCuAC	S	Copper chaperone PCu(A)C	1
PDDEXK_4	S	PD-(D/E)XK nuclease superfamily	1
PKD,Tannase	C	PFAM blue (type 1) copper domain protein	1
Prok-JAB	S	Prokaryotic homologs of the JAB domain	1
PrpF	P	protein conserved in bacteria	1
Radical_SAM	C	Elongator protein 3, MiaB family, Radical SAM	1
Radical_SAM,TRAM,UPF0004	J	Catalyzes the methylthiolation of an aspartic acid residue of ribosomal protein S12	1
RloB	S	RloB-like protein	1
RraA-like	H	Catalyzes the aldol cleavage of 4-hydroxy-4-methyl-2- oxoglutarate (HMG) into 2 molecules of pyruvate. Also contains a secondary oxaloacetate (OAA) decarboxylase activity due to the common pyruvate enolate transition state formed following C-C bond cleavage in the retro-aldol and decarboxylation reactions	1
SCO1-SenC	S	SCO1/SenC	1
SIR2	K	NAD-dependent lysine deacetylase and desuccinylase that specifically removes acetyl and succinyl groups on target proteins. Modulates the activities of several proteins which are inactive in their acylated form	1
TauD	Q	Taurine catabolism dioxygenase TauD, TfdA family	1
Tautomerase_2	S	tautomerase	1
TetR_C_13,TetR_N	K	transcriptional regulator	1
TetR_N	K	Bacterial regulatory proteins, tetR family	1

ThiF	H	ThiF family	1
Thiolase_C,Thiolase_N	I	Acetyl-CoA acetyltransferase	1
Thioredoxin	O	OST3 / OST6 family, transporter family	1
Thymidylate_kin	F	Phosphorylation of dTMP to form dTDP in both de novo and salvage pathways of dTTP synthesis	1
Transposase_mut	L	Transposase	1
tRNA_anti-like	S	tRNA_anti-like	1
UvrD-helicase,UvrD_C	L	UvrD-like helicase C-terminal domain	1
Z1	L	Z1 domain	1

References for Supplementary Information

1. L. R. Hmelo, *et al.*, Precision-engineering the *Pseudomonas aeruginosa* genome with two-step allelic exchange. *Nat Protoc* **10**, 1820–1841 (2015).
2. A. D. Farr, C. Vasileiou, P. A. Lind, P. B. Rainey, An extreme mutational hotspot in nlpD depends on transcriptional induction of rpoS. *PLoS Genet* **21**, e1011572 (2025).
3. R. O. Schlechter, *et al.*, Chromatic bacteria – A broad host-range plasmid and chromosomal insertion toolbox for fluorescent protein expression in bacteria. *Front. Microbiol.* **9**, 3052 (2018).
